# Adjuvant hyperthermic intraperitoneal chemotherapy (HIPEC) in patients with colon cancer at high risk of peritoneal carcinomatosis; the COLOPEC randomized multicentre trial

**DOI:** 10.1186/s12885-015-1430-7

**Published:** 2015-05-24

**Authors:** Charlotte E L Klaver, Gijsbert D Musters, Willem A Bemelman, Cornelis J A Punt, Victor J Verwaal, Marcel GW Dijkgraaf, Arend GJ Aalbers, Jarmila DW van der Bilt, Djamila Boerma, Andre JA Bremers, Jacobus WA Burger, Christianne J Buskens, Pauline Evers, Robert J van Ginkel, Wilhelmina MU van Grevenstein, Patrick HJ Hemmer, Ignace HJT de Hingh, Laureen A Lammers, Barbara L van Leeuwen, Wilhelmus JHJ Meijerink, Simon W Nienhuijs, Jolien Pon, Sandra A Radema, Bert van Ramshorst, Petur Snaebjornsson, Jurriaan B Tuynman, Elisabeth A te Velde, Marinus J Wiezer, Johannes HW de Wilt, Pieter J Tanis

**Affiliations:** 1 Department of surgery, Academic Medical Centre, University of Amsterdam, Post box 22660, 1105AZ Amsterdam, The Netherlands; 2 Department of oncology, Academic Medical Centre, University of Amsterdam, Post box 22660, Amsterdam, The Netherlands; 3 Department of Surgery, Antoni van Leeuwenhoek hospital/the Netherlands Cancer Institute, Amsterdam, The Netherlands; 4 Clinical Research Unit, Academic Medical Centre, University of Amsterdam, Post box 22660, 1105AZ Amsterdam, The Netherlands; 5 Department of surgery, St. Antonius Hospital, Post box 2500, 3430 EM Nieuwegein, The Netherlands; 6 Department of surgery, Radboud University Medical Centre, Geert Grooteplein-Zuid 22, 6525 GA Nijmegen, The Netherlands; 7 Department of surgery, Erasmus Medical Centre/Daniel den Hoed, Post box 2040, 3000 CA Rotterdam, The Netherlands; 8 Dutch Cancer Patient Organization ‘Leven met Kanker’, Utrecht, the Netherlands; 9 Department of surgery, University Medical Centre, Hanzeplein 1, 9700 RB Groningen, The Netherlands; 10 Department of surgery, University Medical Centre, Post box 85500, 3584 CX Utrecht, The Netherlands; 11 Department of surgery, Catharina Ziekenhuis, Post box 1350, 5602 ZA Eindhoven, The Netherlands; 12 Department of pharmacy, Academic Medical Centre, University of Amsterdam, Post box 22660, 1105AZ Amsterdam, The Netherlands; 13 Departement of surgery, Vrije University Medical Center, Post box 7057, 1007 MB Amsterdam, The Netherlands; 14 Society of patients with cancer of the gastrointestinal tract (SPKS), Darmkanker Nederland, Utrecht, the Netherlands; 15 Department of oncology, Radboud University Medical Centre, Geert Grooteplein-Zuid 22, 6525 GA Nijmegen, The Netherlands; 16 Department of pathology, Antoni van Leeuwenhoek hospital/the Netherlands Cancer Institute, Amsterdam, The Netherlands

**Keywords:** Adjuvant hyperthermic intraperitoneal chemotherapy (HIPEC), Colon cancer, Peritoneal carcinomatosis (PC)

## Abstract

**Background:**

The peritoneum is the second most common site of recurrence in colorectal cancer. Early detection of peritoneal carcinomatosis (PC) by imaging is difficult. Patients eventually presenting with clinically apparent PC have a poor prognosis. Median survival is only about five months if untreated and the benefit of palliative systemic chemotherapy is limited. Only a quarter of patients are eligible for curative treatment, consisting of cytoreductive surgery and hyperthermic intraperitoneal chemotherapy (CR/HIPEC). However, the effectiveness depends highly on the extent of disease and the treatment is associated with a considerable complication rate.

These clinical problems underline the need for effective adjuvant therapy in high-risk patients to minimize the risk of outgrowth of peritoneal micro metastases. Adjuvant hyperthermic intraperitoneal chemotherapy (HIPEC) seems to be suitable for this purpose. Without the need for cytoreductive surgery, adjuvant HIPEC can be performed with a low complication rate and short hospital stay.

**Methods/Design:**

The aim of this study is to determine the effectiveness of adjuvant HIPEC in preventing the development of PC in patients with colon cancer at high risk of peritoneal recurrence. This study will be performed in the nine Dutch HIPEC centres, starting in April 2015. Eligible for inclusion are patients who underwent curative resection for T4 or intra-abdominally perforated cM0 stage colon cancer. After resection of the primary tumour, 176 patients will be randomized to adjuvant HIPEC followed by routine adjuvant systemic chemotherapy in the experimental arm, or to systemic chemotherapy only in the control arm. Adjuvant HIPEC will be performed simultaneously or shortly after the primary resection. Oxaliplatin will be used as chemotherapeutic agent, for 30 min at 42-43 °C. Just before HIPEC, 5-fluorouracil and leucovorin will be administered intravenously. Primary endpoint is peritoneal disease-free survival at 18 months. Diagnostic laparoscopy will be performed routinely after 18 months postoperatively in both arms of the study in patients without evidence of disease based on routine follow-up using CT imaging and CEA.

**Discussion:**

Adjuvant HIPEC is assumed to reduce the expected 25 % absolute risk of PC in patients with T4 or perforated colon cancer to a risk of 10 %. This reduction is likely to translate into a prolonged overall survival.

**Trial registration number:**

NCT02231086 (Clinicaltrials.gov)

## Background

Colorectal cancer (CRC) is the third most common cancer in the world. In 2012, almost 1.4 million new patients were diagnosed, with an expected incidence of 2.4 million in 2035 [1]. The peritoneum is the second most common site of recurrence in patients with CRC, accounting for 25 to 35 % of all recurrences [2, 3]. Because the clinical diagnosis of peritoneal carcinomatosis (PC) is much more difficult than the diagnosis of liver or lung metastases, it is likely that reported incidences of metachronous PC are underestimated. Important risk factors for peritoneal tumour seeding of CRC identified in the literature are advanced stage of the primary tumour (pT4) and tumour perforation [4–7].

**Fig. 1:**
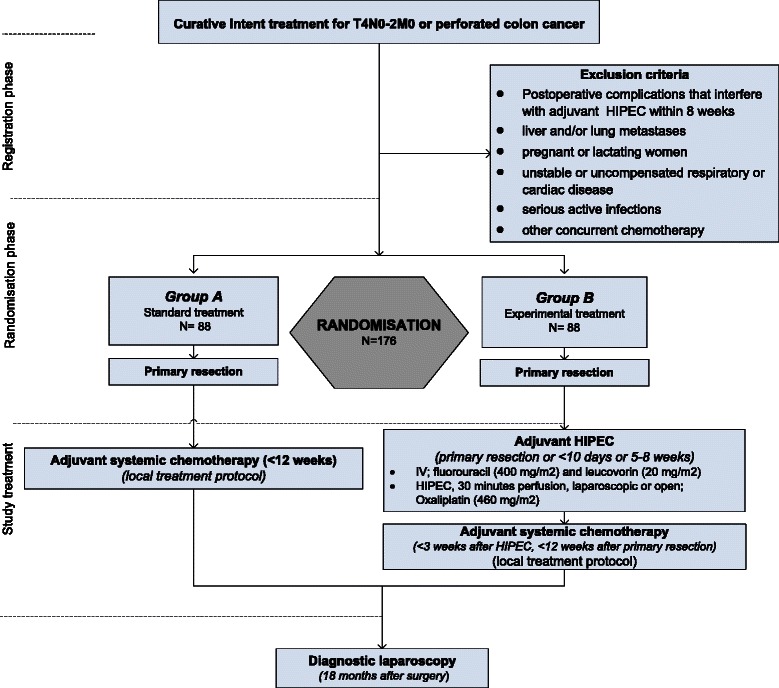
Flow-diagram COLOPEC trial. HIPEC: hyperthermic intraperitoneal chemotherapy

PC of colorectal cancer origin is associated with a poor prognosis. Median survival is only about 5 months if untreated and has a reported range between 5 and 15 months if treated with palliative systemic therapy, being significantly worse compared to survival rates after palliative systemic therapy for non-peritoneal localizations [8–11]. Quality of life is often significantly impaired because of ascites and bowel obstruction [11]. In three quarters of the patients with PC of colorectal origin, only palliative treatment options remain at time of diagnosis [12]. In the remaining quarter of the patients without distant metastases and restricted peritoneal tumour load, cytoreductive surgery (CR) and HIPEC is an intentionally curative treatment option. A large number of phase II studies and two phase III trials have been published on CR/HIPEC, showing an improved survival in comparison with systemic chemotherapy only [8, 13–24]. However, the effectiveness of CR/HIPEC highly depends on the extent of disease. If complete cytoreduction of PC is obtained, 5-year survival rates of 45 to 51 % can be achieved in combination with HIPEC, but survival is significantly lower if not all visible tumour could be resected [25, 26]. Furthermore, CR/HIPEC is associated with substantial morbidity, namely infectious complications and abdominal wall complications.

Because of the difficulties in treating PC at a clinically overt stage and the restricted sensitivity of imaging modalities to detect PC at an early stage, advancing the treatment to a subclinical stage may overcome the current problems in treating patients with PC of colorectal origin. In other words, effective adjuvant treatment to prevent development of PC in high-risk CRC patients is warranted.

Intraperitoneal administration of chemotherapy has been used to treat or prevent PC from various primary malignancies [19, 27–34]. From a pharmacological point of view, this is an attractive approach given the peritoneal-plasma barrier, which allows for higher peritoneal cavity concentrations resulting in higher efficacy while systemic toxicity is not increased.

In an attempt to prevent PC of colorectal origin, intraperitoneal 5-FU administration (IPEC) through a peritoneal catheter in the immediate postoperative period or as prolonged treatment up to 12 months has been used, as well as HIPEC using mitomycin-C or oxaliplatin [31, 32, 35–38]. It can be concluded from these studies that intraperitoneal chemotherapy seems to reduce intraperitoneal recurrence rates, and that even a survival benefit is suggested in studies using adjuvant HIPEC [39]. These studies are subjected to significant bias and no definitive conclusions can be drawn based on these data. With regard to treatment-related morbidity of adjuvant (H)IPEC, these data, as well as experience from the first 10 patients included in a Dutch feasibility study, reveal that adjuvant HIPEC is a well-tolerated intervention with no significant morbidity, which can be performed in a short stay setting [39, 40]. This supports conducting a randomized trial to determine the oncological effectiveness of adjuvant HIPEC in addition to routine adjuvant systemic therapy.

## Methods/Design

### Objective

The primary aim of this study is to determine the oncological effectiveness of adjuvant HIPEC using oxaliplatin, following a curative resection of a T4 or intra-abdominally perforated colon carcinoma in preventing the development of PC.

Secondary aims are:

1. to determine the incidence of PC in pT4 and perforated colon cancer with metastatic patterns based on prospectively collected data and diagnostic laparoscopy at 18 months as a golden standard during follow-up;
2. to identify molecular parameters in tissues of primary tumours indicating high-risk of developing PC;
3. to determine treatment-related morbidity of open and laparoscopic adjuvant HIPEC;
4. to determine treatment-related morbidity of simultaneous and staged adjuvant HIPEC (both early postoperative (0–10 days) as well as delayed (5–8 weeks));
5. to determine several procedural characteristics of adjuvant HIPEC such as operating time, hospital stay, and re-admission rate;
6. to compare quality of life and costs of adjuvant HIPEC with standard adjuvant systemic treatment.

### Design

This will be a randomized controlled clinical trial of the Dutch Colorectal Cancer Group (DCCG) that will be performed in the nine Dutch HIPEC centres, starting in April 2015. Eligible patients will be randomized (in a 1:1 ratio) to adjuvant HIPEC followed by standard adjuvant systemic chemotherapy in the experimental arm, or adjuvant systemic chemotherapy alone in the control arm (Fig. 1). Stratification factors will be tumour characteristic (T4 or perforation), surgical approach of the primary tumour resection (laparoscopy or open) and age (<65 years or ≥65 years).

Timing of adjuvant HIPEC is tailored to the different clinical entities within the study population. Patients with obvious clinical T4 stage can be asked preoperatively for informed consent with simultaneous HIPEC if randomized in the experimental arm. If patients are randomized postoperatively based on intra-operative findings, early staged adjuvant HIPEC is preferably performed as soon as possible after primary resection but at a maximum of 10 days. When early postoperative HIPEC is not feasible, delayed staged adjuvant HIPEC will be performed in week 5 to 8 from primary resection. Adjuvant HIPEC can be performed either by laparoscopy or open approach. Subsequently, patients will receive routine adjuvant systemic chemotherapy according to local treatment protocols within 3 weeks from HIPEC, preferably as soon as the clinical condition allows for systemic therapy. Follow-up will be performed routinely according to the national guideline during the first 18 months. Patients who already developed recurrent disease during this time interval will be treated accordingly. During the entire postoperative period, concomitant medications, adverse events, procedures and adjuvant therapies will be reviewed and documented. In patients who have no clinical signs of recurrent disease at 18 months on CT scan of the thorax and abdomen in combination with non-elevated CEA levels, diagnostic laparoscopy will be performed in both arms of the study. Laparoscopy enables accurate assessment of the primary endpoint of the study and may have therapeutic implications for patients in whom asymptomatic PC is proven by laparoscopically taken biopsies. These patients will subsequently be treated by CR/HIPEC according to the national guideline if fulfilling the treatment criteria, with a switch to mitomycin-C in patients who underwent adjuvant HIPEC with oxaliplatin previously. The staging laparoscopy at 18 months is supported by the increasing data in the literature on the value of second look surgery for high-risk patients [41, 42]. Patients with a negative laparoscopy will continue routine follow-up for at least 5 years from primary resection and during this period, oncological outcome in terms of local recurrence, metastases and survival as well as oncological therapies will be documented. All relevant data during work up, management and follow up will be collected in an electronic case record form. In addition, quality of life and economic evaluation questionnaires will be administered to the patient at each follow-up interval. Data will be documented in line with ‘Good Clinical Practice’ and Dutch legal requirements.

### Study population

Patients diagnosed with adenocarcinoma of the colon and either one of the two following risk factors for PC or both will be considered for inclusion:

- T4N0-2M0, either consisting of obvious clinical T4 stage based on preoperative imaging or intraoperative findings, or pathological T4 stage;
- primary tumour presenting with perforation being curatively resected (N0-2 M0 stage).

Those patients are eligible for this study when they meet the following inclusion criteria:

- age between 18 and 75 years;
- intention to start adjuvant systemic therapy;
- adequate clinical condition to undergo simultaneous HIPEC, or re-laparoscopy/laparotomy with HIPEC within either 10 days or between week 5–8 from primary resection;
- white blood cell count of at least 3000/mm3, platelet count of at least 100.000/mm3;
- normal creatinine or creatinine clearance of at least 50 ml/min;
- written informed consent.

A potential subject who meets any of the following criteria will be excluded from participation in this study:

- postoperative complications that interfere with adjuvant HIPEC within 8 weeks (i.e. persisting intra-abdominal abscess, significant fascial dehiscence, enteric fistula);
- non-curative intent of treatment;
- liver and/or lung metastases;
- pathological T4N0M0 stage with microsatellite instability;
- unstable or uncompensated respiratory or cardiac disease;
- severe hepatic or renal dysfunction;
- bleeding diathesis or coagulopathy.

### Treatment strategies

#### Standard care of the control arm

Treatment in the control arm of the COLOPEC trial is in accordance with the Dutch guideline for adjuvant chemotherapy in colon cancer patients (www.oncoline.nl). First line adjuvant systemic chemotherapy for colon cancer consists of six months treatment with capecitabin and oxaliplatin (CAPOX) every three weeks or 5-FU and oxaliplatin (FOLFOX) every two weeks. Adjuvant chemotherapy is preferably started within six to eight weeks after primary surgery and at a maximum of 12 weeks after primary resection.

#### Investigational treatment of the experimental arm

Treatment in the experimental arm consists of adjuvant HIPEC followed by standard systemic chemotherapy. HIPEC can be performed either by a laparoscopic or open approach to the discretion of the surgeon.

1. *Laparoscopic adjuvant HIPEC*
  Minimally invasive access to the abdominal cavity is obtained, followed by adhesiolysis if indicated and thorough inspection of the peritoneal surfaces. At least one multiperforated inflow catheter is placed through a 10–12 mm port in Douglas pouch with at least one multiperforated outflow catheter in the right subphrenic space. The patient’s body temperature will be monitored in the oesophagus. All trocars are tightly fixed to the skin to avoid fluid leakage during the procedure. Perfusion by an auto regulated pump system will be started with a minimum of 2 L isotonic dialysis fluid at a flow rate of 1-2 L/min and an inflow temperature of 42-43 °C. Before the beginning of HIPEC, fluorouracil 400 mg/m2 and leucovorin 20 mg/m2 will be administered intravenously to potentiate oxaliplatin activity. After attaining at least 42 °C inflow temperature, oxaliplatin (460 mg/m2) will be added to the circuit in a single dose. The operating table will be rotated and tilted every 5 min, and the abdomen will be agitated throughout the infusion to allow homogeneous exposure of the peritoneal surfaces to the heated chemotherapy. After a total perfusion time of 30 min, the peritoneal fluid is totally suctioned and the abdomen is examined for evidence of tissue injury or bleeding. A suction drain will be left in Douglas pouch for 24 h. The other port sites are closed in a standard fashion. Postoperative care after simultaneous HIPEC will be according to the primary colonic resection following an enhanced recovery program. After staged laparoscopic HIPEC, patients are fully mobilized at day one with normal diet and will intentionally be discharged at day one to three if the institutional discharge criteria are fulfilled. Hematologic parameters will be determined at day 14, followed by start of systemic chemotherapy.
2. *Open adjuvant HIPEC*
  Open adjuvant HIPEC can be performed simultaneously in patients undergoing primary open resection, and staged open adjuvant HIPEC can be performed by re-laparotomy in patients who underwent primary open CRC resection. The decision to perform staged open or laparoscopic HIPEC in case of prior open resection will be left to the discretion of the surgeon. Besides the access to the peritoneal cavity, the procedure is similar to the laparoscopic approach as described above. Preferably, a closed perfusion is performed rather than a Colosseum technique to have similar pharmacokinetics as a laparoscopic approach. After positioning of the in- and outflow catheters, the abdomen will then be closed and subsequently perfusion will be started. Postoperative care is similar to the laparoscopic approach with an anticipated day of discharge between day two to five if discharge criteria are fulfilled. Hematologic parameters will be determined at day 14, followed by start of systemic chemotherapy.

### Main outcome

The primary endpoint of the study is peritoneal recurrence-free survival at 18 months determined by CT and if negative by laparoscopy.

#### Secondary study endpoints

- treatment-related toxicity of adjuvant HIPEC, including 30-day complication rate, re-intervention rate, and re-admission rate;
- hospital stay for simultaneous and staged HIPEC, either open or laparoscopic;
- 3 and 5-year disease-free survival and overall survival;
- quality of life (EORTC-30, EORTC-CR29);
- costs (general, per year free of PC, per quality adjusted life year) (EQ-5D-5 L, iMTA MCQ and iMTA PCQ, adapted to the current study setting and target population).

#### Additional outcome measures

- incidence of PC;
- sensitivity of imaging to detect PC during follow-up, using laparoscopy as a golden standard;
- inter- and intra-variability amongst radiologists in detecting PC using CT-imaging;
- differences in patterns of dissemination (peritoneal plus or minus distant metastases);
- impact of adjuvant HIPEC on the degree of adhesions and extent of adhesiolysis required at 18 months;

### Sample size calculation

Approximately 25 % of CRC patients with a pT4 or perforated primary tumour is expected to develop PC (see below). Adjuvant HIPEC is expected to result in a 60 % relative risk reduction in peritoneal recurrence based on the currently available literature (see below). To detect an absolute 15 % difference in PC recurrence-free survival at 18 months (90 % peritoneal recurrence-free under HIPEC plus systemic chemotherapy against 75 % peritoneal recurrence free under systemic chemotherapy), a total number of 176 patients (88 in each arm) is needed (Kaplan-Meier, one-sided, alpha = 0.05, power of 80 %, drop-out 5 %). Actually, the power may even be higher, because the calculation ignores longer follow-up for the patients who have been included early on in the study.

### Data-analysis

The primary endpoint, peritoneal recurrence-free survival at 18 months, will be compared between the two study groups, using Kaplan Meier survival analysis with log rank test and a significance level of 0.05.

#### Secondary study endpoints

Treatment effects will be expressed as a relative risk with 95 % confidence interval. Any binary secondary outcome measures (e.g. re-operation rate, mortality rate, etc.) will be analysed by using a Fisher’s exact or Chi-square test with a two-sided significance level of 0.05 on an intention to treat basis. Continuous variables will be analysed by independent samples *t*-test. Quality of life data will be assessed using the questionnaires ‘EORTC-QLQ-C30’ and ‘CR 29’. The EORTS-QLQ-C30 is a questionnaire developed to test global quality of life in cancer patients. In addition, the EORTC-QLQ-CR29 focusses on colorectal cancer patients. Results will be graphically represented across all time points and analysed using a repeated measures analysis of variance. All analyses will be intention to treat. A p-value of <0.05 will be considered statistically significant. Subgroup analyses will employ a test of interaction to explore whether there is evidence that the treatment effects differ across subgroups. As with all subgroups analyses, these will be interpreted with caution, and will be considered hypothesis generating.

#### Economic evaluation

The economic evaluation of adjuvant HIPEC followed by systemic chemotherapy against adjuvant systemic chemotherapy alone will be performed from the societal perspective as a cost-effectiveness as well as cost-utility analysis. The primary outcomes will be the costs per year free of PC and the costs per quality adjusted life year (QALY) respectively. Incremental cost-effectiveness and cost-utility ratios will be calculated for these outcomes, along with 95 % confidence intervals based on non-parametric bootstrapping to account for sampling variation. Explorative subgroup analyses will be done for patients presenting with or without perforation and for patients with early (simultaneous/<10 days postoperatively) or delayed (5–8 weeks postoperatively) HIPEC. Sensitivity analyses will be performed for the unit costs of adjuvant HIPEC and for alternative health valuation algorithms (see below). Results will be displayed graphically with cost-effectiveness planes and cost-effectiveness acceptability curves for willingness-to-pay values up to €100,000.

The cost analysis will include all direct and indirect medical and non-medical costs. Data on volumes will be gathered from case reports forms, hospital information systems and with short patient questionnaires at quarterly intervals during follow-up. The iMTA Medical Consumption Questionnaire and iMTA Productivity Cost Questionnaire, adapted to the current study setting and target population, will be used.

Dissemination of cancer to the peritoneum may debilitate a person’s quality of life on top of recovering from surgery for the primary cancer. In addition to the already mentioned cancer specific EORTC-QLQ-C30 and CR 29 questionnaires, patients will be asked to complete the short EQ-5D-5L (Euroqol) generic health status questionnaire at each follow-up time point in order to gather health status profiles over time that can be transposed into QALYs using health utility scoring algorithms available from the literature [43, 44] These algorithms reflect preferences in the general population, which were elicited with time trade-off elicitation techniques.

Also, a budget impact analysis (BIA) will be performed linking data on disease incidence and prevalence, on inclusion criteria, and on health care expenses per case. Both governmental and health care insurer perspectives will be addressed.

### Safety

The medical ethical committee of the Academic Medical Center Amsterdam has approved the study protocol (MEC 2014–264, NL49960.018.14) and this study will be conducted according to the principles of the Declaration of Helsinki (Fortaleza, October 2013) and in accordance with the Medical Research Involving Human Subjects Act (WMO). An interim review will be performed after inclusion of 25, 50 and 100 (of the total of 176 patients). At 6 weeks after inclusion of these patients the trial’s safety data will be evaluated. The data and safety monitoring board (DSMB) will be supplied with the number of (serious) adverse events in both groups at this time point. If there is a skewed distribution of the number of (serious) adverse events between the two groups an efficacy analysis can be performed at the discretion of the DSMB. Following these interim analyses the DSMB will advise upon continuation of the trial.

## Discussion

### Rationale for HIPEC design; chemotherapeutic agent, surgical approach and timing

In the COLOPEC trial we will use oxaliplatin as standard chemotherapeutic agent for HIPEC. In the Netherlands, we have recently switched from mitomycin-C as the standard agent for HIPEC to oxaliplatin in all HIPEC centres. Oxaliplatin and mitomycin-C are both cell cycle independent alkylating agents, interfering with DNA and DNA-synthesis. Because of a large molecular weight, there is limited systemic absorption of both agents. The enhancement of cytotoxicity under hyperthermia and a maximal tissue penetration of 2 mm. are also comparable. Although there are no randomized studies comparing oxaliplatin and mitomycin-C for CR/HIPEC, the literature suggests an equal antitumor effectiveness [45]. The advantage of oxaliplatin is the absence of neutropenia and shorter perfusion time (30 versus 90 min.) compared to mitomycin-C.

In this project, there is the possibility to deliver HIPEC by both the conventional open approach and a minimally invasive approach. If compared to an open procedure, laparoscopic HIPEC avoids the risk and recovery time associated with a laparotomy while the temperature profiles and peritoneal perfusion flow rates are similar [46]. Experimental studies in pigs suggest even better penetration of chemotherapy in a closed abdomen probably due to increased abdominal pressure [47, 48].

Prophylactic resection of organs at risk of harbouring tumour cells, such as omentectomy and ovariectomy, has been performed in addition to adjuvant HIPEC [49]. There is currently no evidence to support this. Because HIPEC is applied in adjuvant setting with the majority of patients not expected to benefit from this intervention, we aim to minimise invasiveness of the therapy. Therefore, we decided not to perform prophylactic omentectomy, ovariectomy or any other prophylactic surgery . Moreover, we hypothesise that potential micro metastases at these sites are sufficiently treated with HIPEC .

Timing of the adjuvant HIPEC procedure will be tailored to the individual patient within the COLOPEC trial. Both a simultaneous and a staged approach can be chosen. A theoretical disadvantage of a staged approach is the suggested phenomenon of residual cancer cells being encapsulated with fibrin, which probably makes these cells less accessible for chemotherapy at an interval of more than two weeks after surgery [20, 50–52]. Therefore, it is often stated that HIPEC should ideally be performed simultaneously with primary tumour resection, although this is not an evidence-based recommendation. Simultaneous adjuvant HIPEC is often not feasible because pT4 stage is only found at definitive pathology, HIPEC is not available at time of primary tumour resection in an emergency setting for a perforated tumour, or primary resection is being performed outside a HIPEC centre. In order to make results from a RCT transferable to daily clinical practice in the end, the possibility of early postoperative HIPEC has been included in the study protocol. Staged HIPEC may be performed within 10 days in case of adequate patient condition and logistics, or may be delayed to 5–8 weeks from primary resection. The theoretical advantage of staged adjuvant HIPEC is that healing of the anastomosis is not compromised by immunosuppression as a result of the administration of chemotherapeutic agents, and the more favourable logistics. Disadvantages of staged adjuvant HIPEC are the necessity of adhesiolysis, the risk of suboptimal distribution of chemotherapy in the abdominal cavity, and the potential delay of routine adjuvant systemic treatment.

### Primary endpoint

Although 3-year disease-free survival (3-years-DFS) using CT imaging is a commonly used endpoint in adjuvant setting, 18 month laparoscopy is more appropriate in this trial because of the restricted sensitivity of imaging modalities to detect PC at an early stage. Also, second look surgery is increasingly applied in patients at high risk of developing PC in- and outside trial setting. Positive findings during laparoscopy may have therapeutic implications, which justifies an invasive method to determine the primary outcome parameter. It is likely that a significant reduction in peritoneal recurrence rate at 18 months will eventually translate into an overall survival benefit given the worse prognosis associated with peritoneal dissemination.

### Expected results and impact on clinical practice and health care system

There needs to be a good balance between associated costs and morbidity on one hand and effectiveness on the other hand for an adjuvant treatment modality. For routine adjuvant systemic chemotherapy in colon cancer, an absolute survival benefit as low as 5 % is considered to be worthwhile despite duration of treatment for six months with treatment related toxicity such as hand-foot syndrome.

Based on reported incidences of PC between 14 and 58 % for perforated tumours [41, 53, 54] and between 17 and 50 % for pT4 stage [55–58], the estimated incidence of PC in the study population will be 25 %. Reported incidences may have been underestimated because of the inaccuracy of routine follow-up examinations to detect PC and incomplete autopsy rates of patients who died of CRC. The relative risk reduction in a comparative study of 25 patients undergoing prophylactic HIPEC compared to a 1:2 matched control group was 82 % [59]. Older randomized studies using intraperitoneal 5-FU showed relative risk reductions of peritoneal recurrence of 78 % [38] and 62 % in favour of the intraperitoneal chemotherapy groups [37]. Based on these data, a conservative expected relative risk reduction of adjuvant HIPEC is 60 %. The expected advantage of the experimental intervention is therefore an absolute reduction of 15 % in PC (from 25 % to 10 %).

As a consequence of a 15 % absolute risk reduction, from each cohort of 100 patients, 85 patients will undergo an additional treatment without any benefit. This is only acceptable if the associated morbidity is relatively low, which appears to be so based on systematic review of the literature and our own feasibility study [39, 40].

With regard to cost-effectiveness, additional costs of routine adjuvant HIPEC should be weighed against gained life years and reduced costs of patients in whom PC has been prevented to develop. A 15 % reduction of peritoneal recurrence is expected to result in at least a 5 % survival benefit, given the dismal prognosis associated with development of PC and based on an update of the study by Sammartino et al. [49]. Reduced costs may be related to less expensive treatment for clinically manifest PC, such as CR/HIPEC (approximately €50.000 per procedure) and palliative systemic treatment including expensive targeted agents such as bevacizumab (€2.400 per month per patient). Furthermore, reduced costs may be related to a reduced use of other palliative treatment modalities (ascites drainage, palliative surgery) and less need for palliative care in a hospital or other institutional setting, or at home.

## Abbreviations

COLOPEC: Adjuvant hyperthermic intraperitoneal chemotherapy in patients with COLon cancer at high risk of PEritoneal Carcinomatosis
CRC: Colorectal cancer
CR: CytoReductive surgery
DALY: Disability adjusted life years
DSMB: Data and Safety Monitoring Board
GCP: Good clinical practice
HIPEC: Hyperthermic IntraPEritoneal Chemotherapy
METC: Medical research ethics committee (MREC) (in Dutch, Medisch Ethische ToetsingsCommissie
PC: Peritoneal carcinomatosis
QUALY: Quality adjusted life year
RCT: Randomized controlled trial
WMO: Medical Research Involving Human Subjects Act (in Dutch: Wet Medisch-wetenschappelijk Onderzoek met mensen
